# Characterization of vitamin D3 biotransformation by the cell lysate of *Actinomyces hyovaginalis* CCASU-A11-2

**DOI:** 10.1186/s13568-024-01694-4

**Published:** 2024-04-24

**Authors:** Ahmad M. Abbas, Walid F. Elkhatib, Mohammad M. Aboulwafa, Nadia A. Hassouna, Khaled M. Aboshanab

**Affiliations:** 1 Department of Microbiology and Immunology, Faculty of Pharmacy, Ain Shams University, African Union Organization St. Abbassia, Abbassia, Cairo 11566 Egypt; 2 Department of Microbiology & Immunology, Faculty of Pharmacy, King Salman International University (KSIU), Ras Sudr, South Sinai, Egypt; 3 Department of Microbiology & Immunology, Faculty of Pharmacy, Galala University, New Galala City, Suez, Egypt

**Keywords:** *Actinomyces hyovaginalis*, Bioconversion, Calcitriol, Cell lysate, Fractionation

## Abstract

A former work conducted in our Lab, lead to in a effective scale up of vitamin D3 bioconversion into calcitriol by *Actinomyces (A.) hyovaginalis* isolate CCASU-A11-2 in Lab fermenter (14 L) resulting in 32.8 µg/100 mL of calcitriol. However, the time needed for such a bioconversion process was up to 5 days. Therefore, the objective of this study was to shorten the bioconversion time by using cell-free lysate and studying different factors influencing bioconversion. The crude cell lysate was prepared, freeze-dried, and primarily fractionated into nine fractions, of which, only three fractions, 50, 100, and 150 mM NaCl elution buffers showed 22, 12, and 2 µg/10 mL, calcitriol production, respectively. Ammonium sulfate was used for protein precipitation, and it did not affect the bioconversion process except at a concentration of 10%w/v. Secondary fractionation was carried out using 80 mL of the 50 mM NaCl elution buffer and the results showed the 80 mL eluent volume was enough for the complete elution of the active protein. The pH 7.8, temperature 28 °C, and 6 h reaction time were optimum for maximum calcitriol production (31 µg/10 mL). In conclusion, the transformation of vitamin D3 into calcitriol was successfully carried out within 6 h and at pH 7.8 and 28 °C using fractionated cell lysate. This process resulted in a 10-fold increase in calcitriol as compared to that produced in our previous study using a 14 L fermenter (32.8 µg/100 mL). Therefore, cell-free lysate should be considered for industrial and scaling up vitamin D3 bioconversion into calcitriol.

## Key points

- Biotransformation of vitamin D into calcitriol has been achieved within 6 h using a cell-free lysate of *A. hyovaginalis* isolate CCASU-A11-2.
- pH 7.8 and incubation temperatures 28 °C were optimum for maximum calcitriol production using the cell-free lysate.
- This study results in a 10-fold (31 µL/10 mL) to that obtained using a 14 L Lab fermenter.
- Bioconversion using cell-free lysate should be considered for industrial production of calcitriol.

## Introduction

Vitamin D3 functions as a fat-soluble prohormone, requiring activation in the liver by the enzyme 25-hydroxylase to obtain 25-hydroxyvitamin D3 (calcidiol). Subsequently, the kidney’s 1α-hydroxylase enzyme hydroxylates calcidiol to yield the fully active form, 1α, 25-dihydroxy vitamin D3 (calcitriol) (DeLuca and Schnoes 1983) (DeLuca 2004). These biologically active forms, especially calcitriol, play a crucial role in normal bone mineralization, and many vital metabolic activities in humans (Abbas et al. 2023). Deficiency in these forms can lead to impaired bone mineralization and various pathological consequences in adults (DeLuca and Schnoes 1983).

Recent reports have highlighted a link between vitamin D3 deficiency and certain immunity disorders, and particular types of cancer(Grant and Giovannucci 2009); He et al. 2016); Al-khalidi et al. 2017); Chung et al. 2017) (Mohajeri Amiri et al. 2019). As a clinical remedy for chronic renal failure, hypoparathyroidism, and osteoporosis, chemically synthesized calcitriol has been used (Grant and Holick 2005) [8]. However, this synthesis, particularly the selective introduction of a hydroxyl group, is a costly and labor-intensive process involving numerous steps (Kametani and Furuyama 1987).

Bioconversion of vitamin D3 offers a more cost-effective alternative to chemical synthesis. Despite this, within the Actinomycetales order, specifically the genera *Streptomyces* and *Amycolata*, only a limited number of microorganisms have demonstrated the capability to conduct this conversion (Sasaki et al. 1991, 1992) (Sawada et al. 2004; Kang et al. 2006) (Takeda et al. 2006). An isolated strain, *A. hyovaginalis* CCASU-A11-2, exhibited the ability to transform vitamin D3 into calcitriol (Abbas et al. 2011a). This was the first documented instance of the genus *Actinomyces* participating in the conversion of vitamin D3 into its physiologically active forms (Abbas et al. 2011a).

Recently, in our Lab we have optimized the biotransformation of vitamin D3 into calcitriol using a laboratory fermenter leading to a 2.5-fold increase to that obtained in the shake flask however, this process takes longer time about 5 days (Abbas et al. 2023). Accordingly, the objective of the current study was to minimize the time needed for the vitamin D3 bioconversion as possible as we can to render the bioconversion process more economical. Therefore, the present work aimed to shorten the time of vitamin D3 conversion into calcitriol by using the free cell lysate for the bioconversion process as well as testing various environmental factors influencing the respective bioconversion process.

## Materials and methods

### Microorganisms, vitamin D3, culture medium

*A. hyovaginalis* CCASU-A11-2 was deposited in the Culture Collection Ain Shams University and was proven to convert vitamin D_3_ into calcitriol (Abbas et al. 2023). It was cultured and preserved as previously reported in our previous study (Miller 1972) (Abbas et al. 2023). Vitamin D3 was generously supplied by Medical Union Pharmaceuticals (MUP), Cairo, Egypt, and calcitriol was procured from Sigma-Aldrich, St. Louis, MO, USA. The culture medium was formulated as previously reported and the pH was adjusted to pH 7.8 (Abbas et al. 2023). The phosphate-buffered salt solution was formulated as previously described (Abbas et al. 2023).

### Preparation of the cell lysate

A single colony of the study isolate from the nutrient agar was inoculated into a 10 mL nutrient broth (seed culture; Oxoid, ThermoFisher Scientific, London, UK) contained in a 100-mL Erlenmeyer flask, incubated at 30 °C and 200 rpm for two days. An aliquot (1 mL) of the obtained seed culture was used to inoculate the 50 mL medium used for the main culture of study isolate contained in a 250 mL Erlenmeyer flask (main culture), incubated at 30 °C and 200 rpm for 48 h. Then centrifugation was carried out at 5000 rpm for 10 min, the sediment was re-suspended in 10 mL phosphate buffered salts solution, and transferred to a 50 mL beaker. The cells were then lysed via sonication using a probe sonicator device adjusted at 70% amplitude as previously reported (Abbas et al. 2011b).

### Primary fractionation of the crude cell lysate

The resultant crude cell lysate suspension, produced above, was then freeze-dried at -50˚C using a laboratory scale freeze-dryer (CoolSafe 55, ScanLaf A/S, Lynge, Denmark) under vacuum at 30 (psi) for 48 h. The powder obtained was dissolved in not more than 2 mL 20 mM Tris-HCl buffer and subjected to fractionation via column chromatography using DEAE–Sepharose CL-6B as a stationary phase and different concentrations (0-400 mM) of NaCl in 20 mM Tris-HCl (pH 7.4), as the mobile phase (Sawada et al. 2004).

The column chromatography process was as follows: stationary phase (50 g) was soaked in 50 mL 20 mM Tris-HCl buffer (pH 7.4) for 24 h, for equilibration. The soaked stationary phase was then packed in a glass column (2 cm inner diameter by 30 cm length) and column conditioning was carried out with 20 mM Tris-HCl (pH 7.4). The sample solution was applied onto the column bed and elution was started using 50 mL of 20 mM Tris-HCl (pH 7.4, containing no NaCl), at a flow rate of 1mL/min. The eluate was collected in a 100-mL Erlenmeyer flask. The elution process was then continued using 50 mL of subsequently increasing concentration of NaCl in 20 mM Tris-HCl (50, 100, 150, 200, 250, 300, 350, and 400 mM NaCl). The eluates obtained for the corresponding NaCl concentrations were separately collected in 100-mL Erlenmeyer flasks.

### Determination of the effect of ammonium sulfate on vitamin D_3_ bioconversion activity of eluted fractions

Proteins were recovered from the collected fractions by the addition of ammonium sulfate at 40% w/v saturation and equilibration for 30 min at 4 °C (Andriani et al. 2012), subsequent centrifugation at 6000 rpm for 10 min, and collection of the residue. The protein residue of each fraction was suspended in 10 mL phosphate buffered salts solution, contained in a 100-mL Erlenmeyer flask, inoculated with 10 mg vitamin D_3_, dissolved in 250 µL 96% ethanol and incubatd at 28 °C for 6 h. The reaction mixtures were extracted and analyzed, for calcitriol production, using HPLC as previously reported (Abbas et al. 2023b).

### Determination of the effect of ammonium sulfate on vitamin D_3_ bioconversion activity of eluted fractions

This was carried out by pooling the fractions with main vitamin D_3_ bioconversion activities into one fraction (100 mL) which was then divided into four aliquots (each of about 25 mL). Protein contents of these aliquots were recovered, by ammonium sulfate precipitation, and treated for testing their vitamin D_3_ bioconversion activities, as previously described, except that:

(і) One aliquot coded A, was subjected to no ammonium sulfate addition.

(іі) The three remaining aliquots, coded A2%, A5%, and A10%, were subjected to ammonium sulfate additions at 2, 5, and 10% w/v concentrations, respectively.

The results obtained were recorded and compared.

### Secondary fractionation

The concentrations of NaCl, in the mobile phase, that eluted fractions with main vitamin D_3_ bioconversion activities were determined. After that, re-fractionation of the crude cell lysate of the study isolate was carried out as previously described, but using mobile phase volumes with NaCl concentrations, corresponding to those of eluted fractions having main vitamin D_3_ bioconversion activities, as follows:

(i) An 80 mL volume of mobile phase, with NaCl concentration corresponding to the lowest concentration that eluted fraction with main vitamin D_3_ bioconversion activity, was applied as multiple aliquots (each of 10 mL).
(ii) A 10 mL volume of mobile phase, with NaCl concentration corresponding to the highest concentration that eluted fraction with main vitamin D_3_ bioconversion activity, was applied once as a single aliquot.
(iii) A 10 mL volume of mobile phase, with NaCl concentration corresponding to that in between the lowest and highest concentrations that eluted fractions with main vitamin D_3_ bioconversion activities, was applied once as a single aliquot.

### Studying the effect of some factors on vitamin D_3_ bioconversion by the chromatographic active fraction

In this experiment, the mobile phase volume required for the complete elution of vitamin D_3_ bioconversion active protein, as determined previously, was subjected to stepwise addition in the form of 10 mL aliquots. Each aliquot was added near finishing the volume of the proceeding one. The eluates, obtained at the end of the applied addition regimen, were collected as a single fraction, (chromatographic active fraction). This fraction was subjected to bioconversion activity measurements, under different reaction conditions.

### Effect of different pH values

The chromatographic active fraction was tested for its vitamin D_3_ bioconversion activity,, under different pH values including 6, 7, 7.5, and 8.5 as previously described by Abbas et al. (2023). The results were compared to those obtained at the pH value 7.8. This experiment was done in triplicate and both the mean and standard deviations were calculated.

### Effect of different reaction temperatures

The chromatographic active fraction was tested for its vitamin D_3_ bioconversion activity, as previously described, under different temperature values bracketing the used temperature value (28 °C). These included 25, 30, and 37 °C. The results were compared to those obtained at 28 °C. This experiment was done in triplicate and both the mean and standard deviations were calculated.

### Effect of different reaction times

The bioconversion reaction was carried out for 6 h. Other reaction times were tested, including 3, 9, and 12 h. The experiments were completed as previously described and the results were compared to those obtained at 6 h. This experiment was done in triplicate and both the mean and standard deviations were calculated.

### Extraction of vitamin D3 and calcitriol

The extraction process was carried out as previously reported (Bligh and Dyer 1959), and (Abbas et al. 2011b). This experiment was done in triplicate and both the mean and standard deviations were calculated.

### High-performance liquid chromatography

The condition and the methods of HPLC analysis was carried out as previously reported (Abbas et al. 2023).

### Measurement of protein concentration

The total protein concentrations, in different experiments, were measured using the Bradford assay (Bradford 1976), using the available commercial reagent (Sigma-Aldrich, St Louis, MO, USA) and following the manufacturer’s manual. The protein concentration was calculated as previously reported (Bradford 1976).

### Statistical analysis

Statistical analysis presented as mean ± Standard Deviation (S.D.) through the use of Excel Microsoft Office 365.

## Results

### Characterization of bioconversion activity of the cell lysate

#### Primary fractionation of the crude cell lysate

**Fig. 1:**
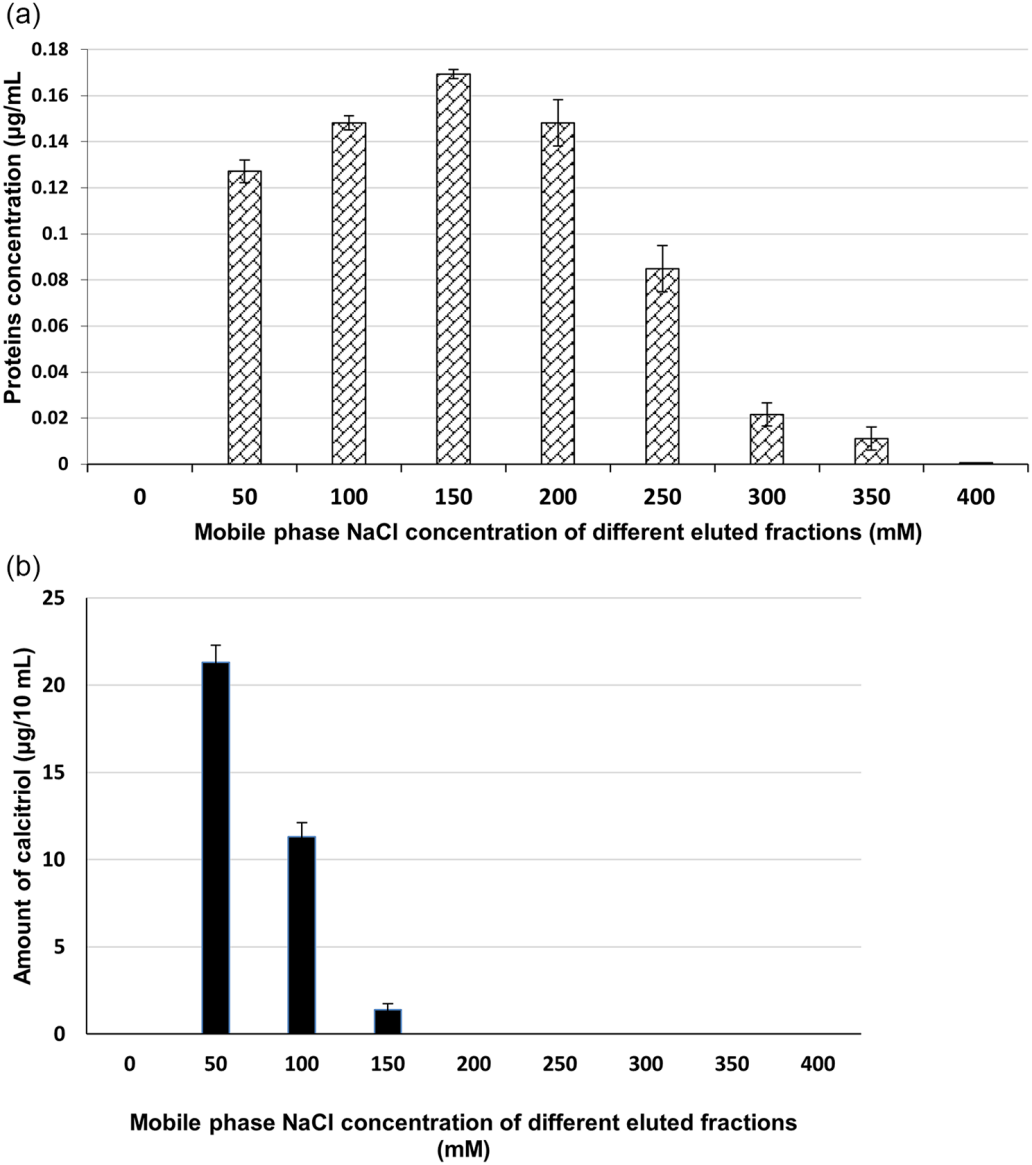
Profiles of proteins concentrations (**a**) as well as vitamin D_3_ bioconversion activity (**b**) of reconstituted reaction mixtures of proteins recovered form different fractions collected from ion exchange chromatographic primary fractionation of the crude cell lysate of *Actinomyces hyovaginalis* isolate CCASU-A11-2. Vitamin D_3_ bioconversion activity was expressed in terms of amounts of calcitriol produced

The crude cell lysate was prepared, freeze-dried, and primarily fractionated, as previously described, using 50 mL aliquots of different concentrations (0-400 mM) of NaCl in 20 mM Tris-HCl (pH 7.4). This resulted in nine fractions, three of which (50, 100, and 150 mM NaCl elution buffers) showed vitamin D_3_ bioconversion activity. Figure 1 illustrates the protein concentrations of each fraction where total protein was measured to be 0.72 µg/mL (Fig. 1a) and vitamin D_3_ bioconversion activity (Fig. 1b) profiles of the collected fractions where only three fractions (50 mL, 100 mL, 150 mL) with a total protein concentration of about 0.45 µg/mL showed bioconversion activity. Accordingly, upon comparing the activity to the total protein in the cell lysate and NaCl fractions, the amount of the produced calcitriol per 1 µg protein (enzyme activity) was 4.86 and 7.8 µg calcitriol/ µg protein (ration of 1:1.6) for the cell lysate and NaCl fractions, respectively.

### Effect of ammonium sulfate on the bioconversion activity of eluted fractions

**Fig. 2:**
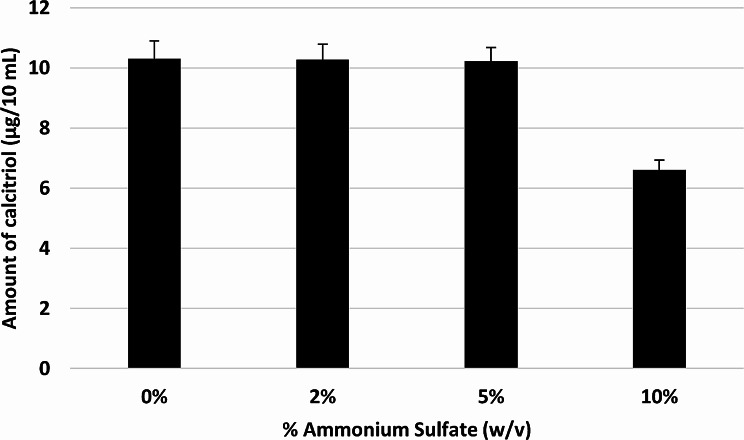
Effect of further ammonium sulfate additions, on vitamin D_3_ bioconversion activities of reconstituted reaction mixtures of un-dialyzed precipitated active protein. Vitamin D_3_ bioconversion activity was expressed in terms of amounts of calcitriol produced

This was carried out to determine the degree of the effect of ammonium sulfate, used for precipitation of vitamin D_3_ bioconversion active protein, on its bioconversion activity. In this experiment, ammonium sulfate was added to the recovered proteins, as previously described. Fortunately, the incorporation of ammonium sulfate, in addition to the residual amount associated with the recovered proteins, has nearly no effect on vitamin D_3_ bioconversion activity of the test fractions, up to 5% w/v addition. However, 10% w/v additional amount of ammonium sulfate decreased the activity by about 35% (Fig. 2). The activity to the total protein in the tested NaCl fraction (100 mL), (enzyme activity) was 7.3 µg calcitriol/ µg protein for the 2 and 5% w/v ammonium sulfate (no effect on the enzyme activity as compared to that obtained in absence of ammonium sulfate) however, the enzyme activity was reduced to 4.7 µg calcitriol/ µg protein upon using 10% w/v ammonium sulfate as depicted in Fig. 2.

### Secondary fractionation

**Fig. 3:**
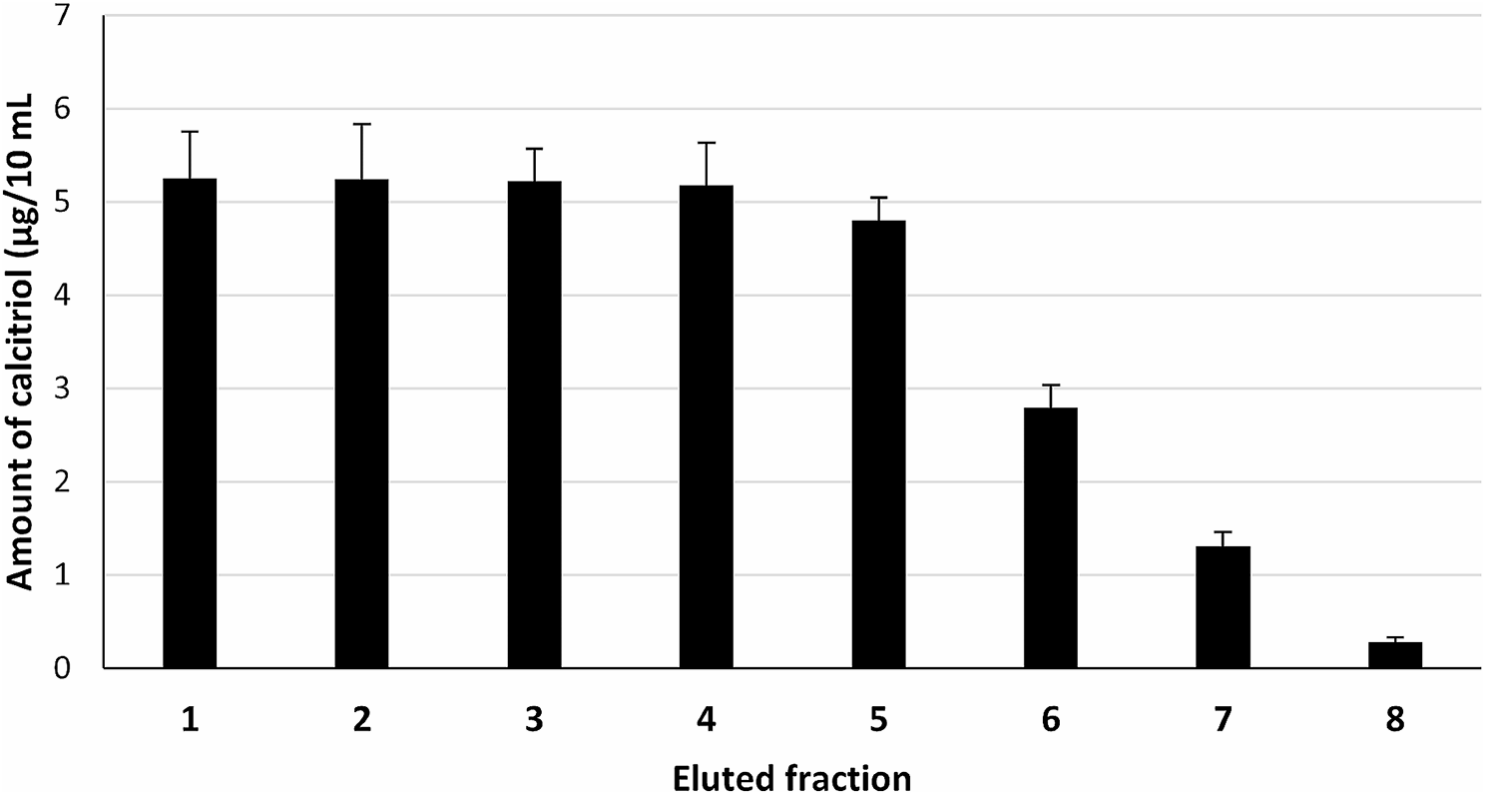
Profiles of vitamin D_3_ bioconversion activity of reconstituted reaction mixtures of protein(s) recovered from different fractions collected from ion exchange chromatographic secondary fractionation, using 50 mM NaCl, of the crude cell lysate of *Actinomyces hyovaginalis* isolate CCASU-A11-2. Vitamin D_3_ bioconversion activity was expressed in terms of amounts of calcitriol produced

In ion exchange chromatographic primary fractionation of the crude cell lysate of the study isolate, vitamin D_3_ bioconversion activity was demonstrated in three 50-mL fractions. The main activity (22 and 12 µg/10 mL, respectively) was obtained in the two fractions eluted by 50 and 100 mM NaCl in 20 mM Tris-HCl. Whereas, a minute activity was obtained in the fraction eluted with 150 mM NaCl in 20 mM Tris-HCl. Subsequently, an additional experiment was carried out to show if vitamin D_3_ bioconversion active protein could be eluted at a single concentration of NaCl (50 mM), by subdividing 80 mL eluent volume into eight fractions (each of 10 mL) instead of using 50 mL eluent volume at once. Further elution at two subsequently higher molar concentrations of NaCl (75 and 100 mM) was carried out, using 10 mL eluent volume of each, for eluting any remaining traces of the active protein (if any). The results revealed that the 80-mL eluent volume of the 50 mM NaCl concentration, appeared to be enough for the complete elution of the active protein since the last fraction showed a minute activity (0.28 µg/10 mL), as illustrated in Fig. 3. This was further confirmed by the absence of any bioconversion activity of proteins eluted by the two 10-mL fractions eluted by higher NaCl concentrations (75 and 100 mM).

### Studying the effect of some factors on vitamin D_3_ bioconversion by the chromatographic active fraction

The chromatographic active fraction, obtained by chromatographic fractionation of the crude cell lysate of the study isolate, was subjected to different factors/conditions that may affect its bioconversion activity. These conditions included different pH values, incubation temperatures, and reaction times.

### Effect of different pH values

**Fig. 4:**
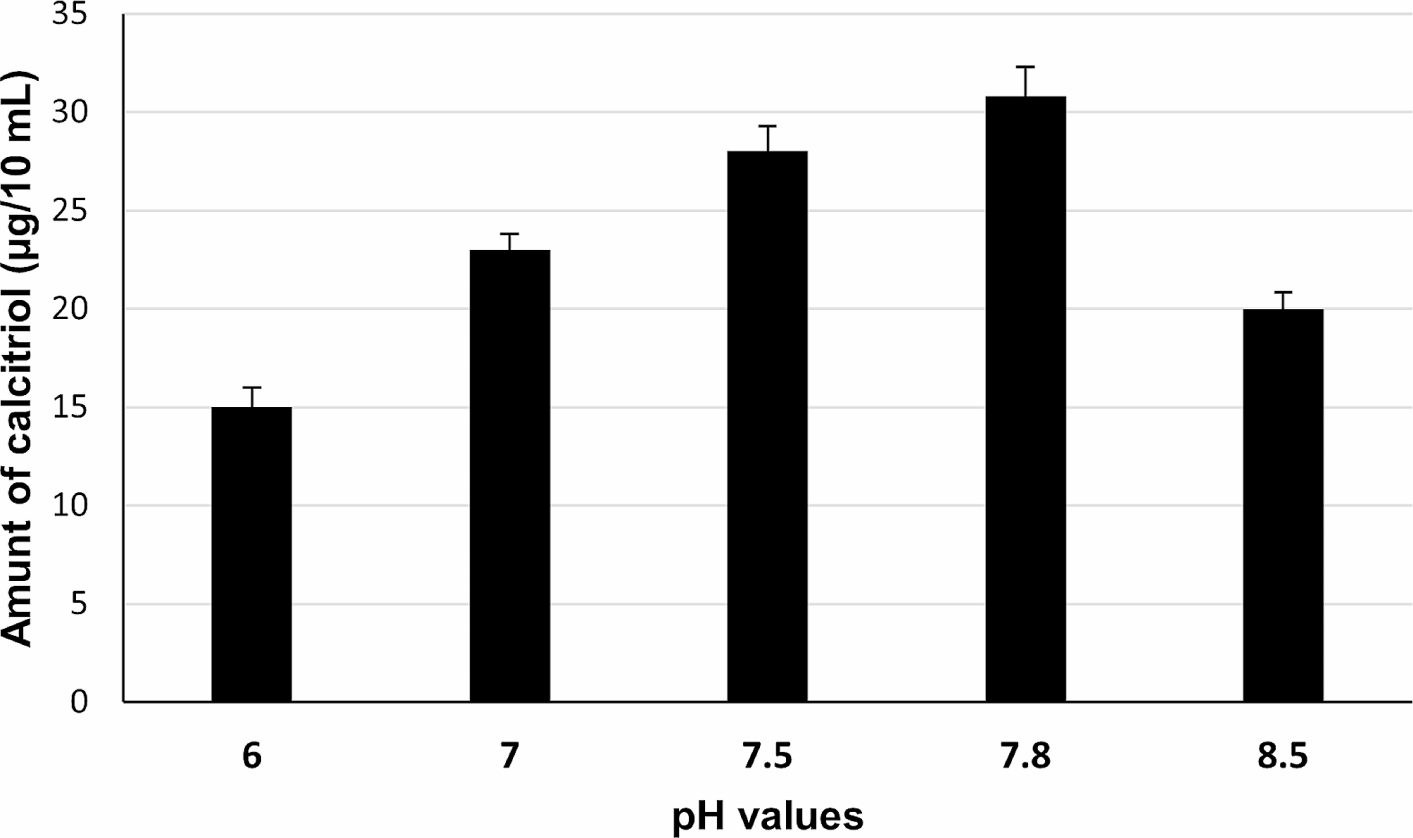
Effect of different pH values on vitamin D_3_ bioconversion activity of reconstituted reaction mixtures of protein(s) recovered from chromatographic active fraction of the crude cell lysate of *Actinomyces hyovaginalis* isolate CCASU-A11-2. Vitamin D_3_ bioconversion activity was expressed in terms of amounts of calcitriol produced. Reaction conditions: reaction temperature of 28 °C and reaction time of 6 h

The chromatographic active fraction was tested for its vitamin D_3_ bioconversion activity under different pH values including 6, 7, 7.5, and 8.5, using a pH value of 7.8 (which was originally applied) as a control. Figure 4 shows that the chromatographic active fraction produced the highest amount of calcitriol (30.8 µg/10 mL) from vitamin D_3_ at a pH value of 7.8.

#### Effect of different reaction temperatures

**Fig. 5:**
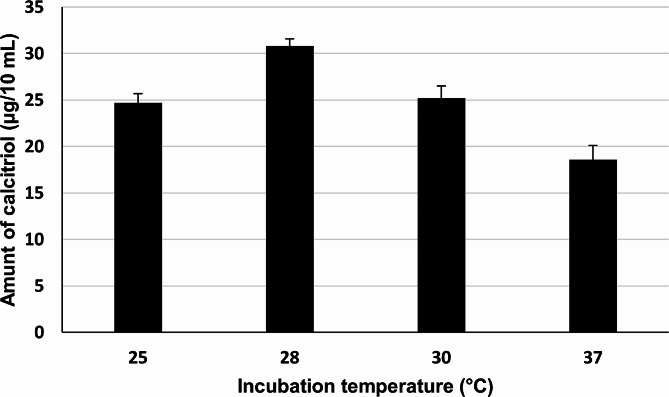
Effect of different reaction temperatures on vitamin D_3_ bioconversion activity of reconstituted reaction mixtures of protein(s) recovered from chromatographic active fraction of the crude cell lysate of *Actinomyces hyovaginalis* isolate CCASU-A11-2. Vitamin D_3_ bioconversion activity was expressed in terms of amounts of calcitriol produced. Reaction conditions: pH of 7.8 and reaction time of 6 h

The chromatographic active fraction was tested for its vitamin D_3_ bioconversion activity under different reaction temperatures including 25, 30, and 37 °C, using a reaction temperature of 28 °C (which was originally applied) as a control. Results revealed that the chromatographic active fraction produced the highest amount of calcitriol (31 µg/10 mL) from vitamin D_3_ when the reaction was incubated at 28 °C, as delineated in Fig. 5.

### Effect of different reaction times

**Fig. 6:**
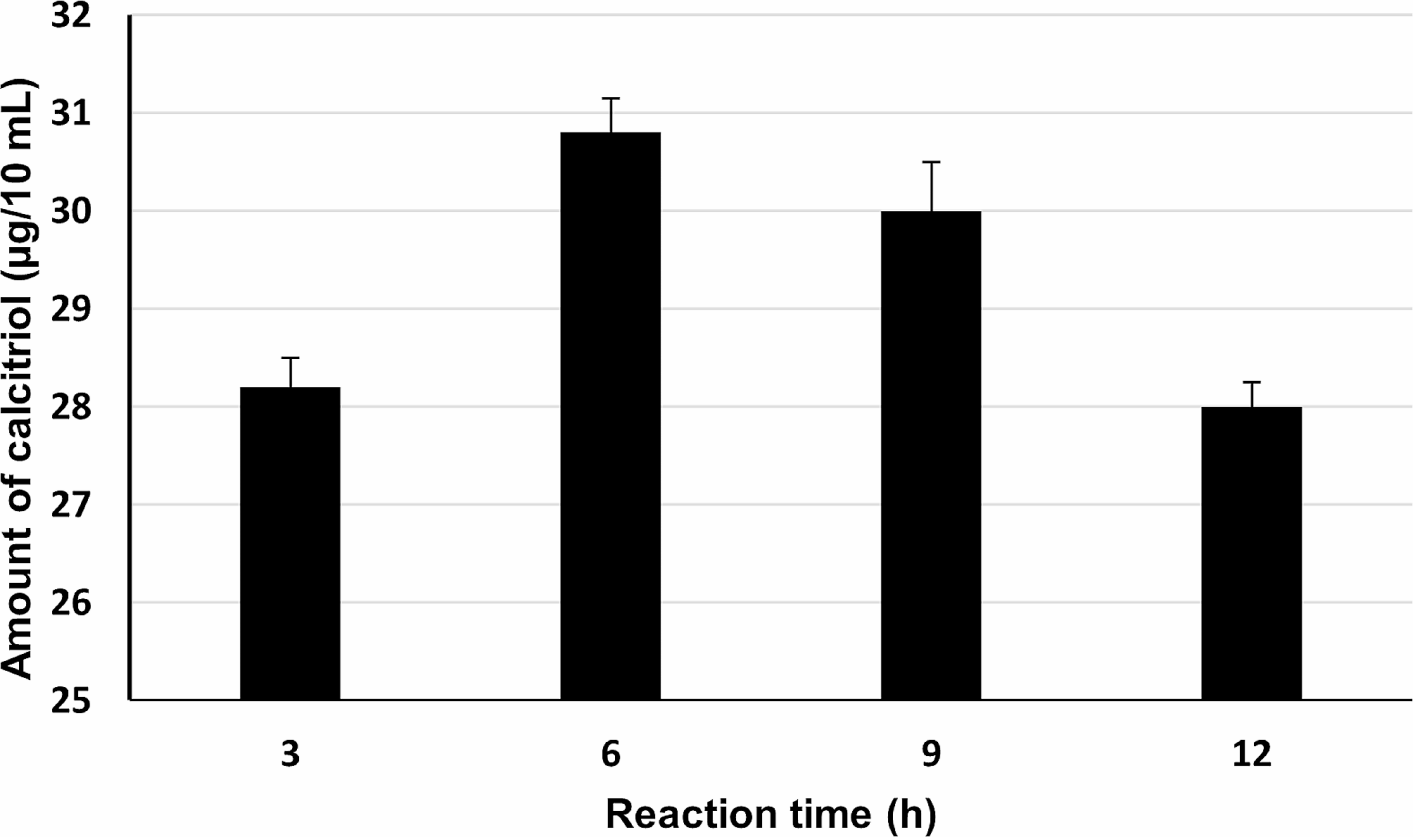
Effect of different reaction times on vitamin D_3_ bioconversion activity of reconstituted reaction mixtures of protein(s) recovered from chromatographic active fraction of the crude cell lysate of *Actinomyces hyovaginalis* isolate CCASU-A11-2. Vitamin D_3_ bioconversion activity was expressed in terms of amounts of calcitriol produced. Reaction conditions: pH of 7.8 and incubation temperature of 28 °C

The bioconversion reaction continued for 6 h. In this experiment, the chromatographic active fraction was tested for its vitamin D_3_ bioconversion activity under different reaction times (3, 9, and 12 h), using a reaction time of 6 h as a control. Results revealed that the chromatographic active fraction produced the highest amount of calcitriol (30.8 µg/10 mL) from vitamin D_3_ when the reaction was continued for 6 h (Fig. 6).

## Discussion

Vitamin D_3_ bioconversion into calcitriol using a laboratory fermenter was accomplished in our lab resulting in a 2.5-fold increase to that obtained in the shake flask however, this process takes a longer time about 5 days (Abbas et al. 2023). As the enzymes involved in such a process are located intracellularly, this resulted in the limitation of the bioconversion process due to diffusion, which can be overcome by using the cell lysate. As a result, vitamin D_3_ bioconversion using different protein fractions of the crude cell lysate as well as studying different environmental factors influencing the bioconversion process, were carried out. Various recent studies have shown the value of the biotransformation processes of certain compounds using various natural lysates to produce valuable bioactive compounds (Taira et al. 2018; Hou et al. 2020a; Zheng et al. 2022; Song et al. 2023) or biodegrade environmental toxic compounds or xenobiotics (Hou et al. 2020b; Zargar et al. 2021).

The fractionation of the crude cell lysate of our study isolate, *A. hyovaginalis* isolate CCASU-A11-2 was carried out by ion exchange chromatography, using anionic resin (DEAE–Sepharose CL-6B) as a stationary phase and different concentrations (0-400 mM) of NaCl in 20 mM Tris-HCl (pH 7.4), as the mobile phase as previously reported (Sawada et al. 2004). The fractionation was carried out primarily to determine the proper concentration and elution volume of the eluent NaCl solution, required for eluting vitamin D_3_ bioconversion active protein. The protein concentration profiles of the collected fractions showed a gradual increase in the eluted protein concentrations reaching the maximum value at 150 mM NaCl concentration followed by a gradual decline to reach the minimum value at 400 mM NaCl concentration. However, it was found that vitamin D_3_ bioconversion activity was limited to three fractions only; two of them (eluted with 50 and 100 mM NaCl concentrations) showed considerable activity, with the 50 mM eluted fraction exhibiting about 2-fold the activity as that of the 100 mM eluted fraction. Whereas, the third fraction, eluted with 150 mM NaCl concentration, showed the lowest activity.

A secondary experiment was carried out to show if vitamin D_3_ bioconversion active protein could be eluted, completely, at the 50 mM concentration of NaCl, by using eight aliquots (each of 10 mL), for elution, instead of using 50 mL eluent volume at once. Further elution at two subsequently higher molar concentrations of NaCl (75 and 100 mM) was carried out, using 10 mL eluent volumes of each, for eluting any remaining active protein (if any). Our results revealed that the 80-mL eluent volume of the 50 mM NaCl concentration, appeared to be enough for the complete elution of the active protein since the last fraction showed a minute activity (0.28 µg/10 mL). This was further confirmed by the absence of any bioconversion activity of proteins recovered from the two fractions eluted by 75 and 100 mM NaCl concentrations. Subsequently, fractions with positive vitamin D_3_ bioconversion activities (eluted by 50 mM concentration of NaCl), were pooled together into a collective fraction, termed (chromatographic active fraction).

Moreover, in this study, the chromatographic eluted proteins were precipitated using ammonium sulfate followed by vitamin D_3_ bioconversion activity measurements, without dialysis. Omission of the dialysis step was applied as it was stated, in some studies, that dialysis of such proteins (those containing vitamin D_3_ bioconversion enzymes) could decrease or abolish the bioconversion activity (Fujii et al. 2009). It is noted that vitamin D_3_ bioconversion activity of the reconstituted reaction mixture of the recovered un-dialyzed proteins, with no ammonium sulfate addition, resulted in an amount of calcitriol of about 10 µg/10 mL. This tested aliquot contained about 5% w/v residual ammonium sulfate in the reconstituted volume, because of the protein precipitation technique applied. Interestingly, a further 5% w/v addition of ammonium sulfate, which nearly increased its level to twice the original one, yielded approximately the same amount of calcitriol (≈ 10 µg/10 mL). This result gives clear evidence that ammonium sulfate, remaining after protein precipitation and oscillating around 5% w/v concentration, does not affect vitamin D_3_ bioconversion activity even when such residual concentration is doubled.

A further experiment was accomplished to study the effect of pH, reaction temperature, and reaction time(Tang et al. 2020) (Wang et al. 2022), on vitamin D_3_ bioconversion activity of the chromatographic active fraction. Our Findings revealed pH 7.8 was optimum for the biotransformation process. Such a finding was similar to that obtained by Kang et al. 2006 and Fujii et al. 2009. Also, it was found that the optimum temperature for the enzyme activity was 28 °C. However, a considerable decline in calcitriol production occurred at 37 °C as previously documented (Kang et al. 2006). It is worth noting that, using the enzymes extracted from cells produced in optimized fermentation, would properly have higher enzyme level and activity as compared to those obtained from in shake flask. Accordingly, one of our future consideration to physiologically and environmentally optimize the cells of *A. hyovaginalis* CCASU-A11-2 in order to produce more active enzymes as previously reported (Fujii et al. 2009; Yasutake et al. 2010).

Our findings regarding the obtained enzymatic activities (4.86 and 7.8 mg calcitriol/mg protein/L (ration of 1:1.6) for the cell lysate and NaCl fractions, respectively are comparable to that performed by Sasaki et al. 1991 using two *Streptomyces* isolates with a conversion productivity of about 7.0 mg/L. Furthermore, Ehrhardt et al. 2016 have used genetically engineered strains to boost the hydroxylation of vitamin D3. Moreover, in another study conducted by Yasutake et al. (2013), where Nisin-treated *Rhodococcus* cells containing VdhT107A was constructed and became able to increase hydroxylation of vitamin D3 to 573 mg/mL in two hours (Yasutake et al. 2013). Another study conducted by Luo et al. (2016), where they used *Pseudonocardia autotrophica* and produced 25 hydroxy-vitamin D3 in a concentration of 639 mg/L but within 120 h. However, in our study, bioconversion of vitamin D into calcitriol has been achieved within 6 h using a cell-free lysate of *A. hyovaginalis* isolate CCASU-A11-2 and the calcitriol production was increased by about 10-fold (31 µL/10 mL) to that obtained using a 14 L Lab fermenter. Therefore, bioconversion using cell-free lysate should be considered for the industrial production of calcitriol production.

Moreover, the maximum calcitriol production was obtained using a reaction time of 6 h. Whereas longer reaction times (9 and 12 h) caused a decline in the amount of calcitriol produced. The obtained results prove that the time of contact of the enzyme with the substrate is a principal reason influencing the bioconversion process. Nevertheless, the fall in the bioconversion process, after 6 h of contact, could be assigned to the mortification of the produced calcitriol. However, our future consideration is to clone the gene coded for vitamin D hydroxylation by our tested isolate and analyze the produced enzyme using SDS-PAGE analysis as well as compare the obtained activity of the recombinant enzyme to that of the crude cell lysate. As previously reported, conversion of vitamin D3 to calcitriol is carried through two sequential enzymatic steps that are mediated by vitamin D3 25-hydroxylase and 25(OH)-vitamin D3 1α-hydroxylase activities to produce calcitriol (Fujii et al. 2009; Yasutake et al. 2010). In this context, Fujii et al. (2009) have succeeded to construct a vitamin D hydroxylase-expressed recombinant strain of *Actinobacterium Rhodococcus erythropolis* which was able to transform vitamin D3 into calcidiol. Moreover, Yasutake et al. (2010) was succeeded to produce recombinant vitamin D3 hydroxylase resulted in enhancement of vitamin D3 hydroxylation activities via the directed evolution of cytochrome P450 vitamin D3 hydroxylase. In conclusion, the biotransformation of vitamin D3 into calcitriol was successfully achieved within 6 h and at pH 7.8 and 28 °C using fractionated cell lysate of *A. hyovaginalis* CCASU-A11-2. This process resulted in a 10-fold increase in calcitriol as compared to that produced in our previous study using a 14 L fermenter (32.8 µg/100 mL) (Abbas et al. 2023). Therefore, using cell-free lysate instead of whole cells should be considered for industrial and scaling up of vitamin D3 bioconversion into calcitriol.

## Data Availability

All data generated or analyzed during this study are present in the main manuscript.
